# Tænicide Properties of Pepo, with Report of a Case in Which It Was Successfully Used

**Published:** 1864-06

**Authors:** E. Ingals


### TÆNICIDE PROPERTIES OF PEPO,

*With Report of a Case in which it was Successfully Used.*

By E. INGALS, M. D.

Pepo—made officinal by the Pharmacopœia of 1860—has been known now for more than a century, to possess properties destructive to the tapeworm. The influence of the doctrine of signatures is said to have first suggested its use for this purpose. Inasmuch as there was observed to be a degree of resemblance between the pumpkin seed and the joints of which the body of the worm was made up, it was surmised that it would be the proper remedy to be used with a view of ridding the system of this troublesome parasite—and as fortune would have it, a doctrine that has its foundation only in ignorance and superstition, was in this instance the parent of truth. But though the pumpkin seed has been so long known as a remedy for tapeworm, yet it has never been extensively employed for this purpose, especially in this country. Its use was introduced into the United States by Dr. J. A. Smith, of Boston, in 1850.

Since that time a considerable number of cases of its successful employment have been reported in our medical journals—such reports, coming mostly from physicians practicing in the Eastern portion of the Republic, and we have reason to think that the remedy has been used there more than at the West or South. The seeds contain a fixed oil, which may be obtained from them by expression, and to this is said to be due their medicinal virtue. Some cases of tapeworm have been successfully treated by the administration of this oil, a fluid ounce being given at one dose, to be followed in two hours by some active cathartic; but the oil is not known to possess any advantages over the seeds in substance.

The case in which we used this remedy occurred in a healthy boy, eleven years old, who, for two months before his parents brought him to me, had been voiding a number of joints of the worm daily, and his previous symptoms, as related to me, justify the conclusion that he had been afflicted with it more than a year, though no treatment had been resorted to, as they did not suspect the nature of the malady. The health of the child did not suffer, except that he was troubled somewhat at night by a cough of a spasmodic nature, and the parents noticed that this was always much worse when the child took milk for his supper, and likewise that prompt relief was sure to follow the taking of some bitter substance. I directed that ℥ j of the pumpkin seeds should be freed from their shells, and then with ʒ iv of sugar and ℥ vj of water, to be made into an emulsion, and of this one-third was to be taken every hour, after fasting from supper until morning, and in one hour after the last dose ℥ ss of castor oil. As this failed to act on the bowels, three hours after, ℥ j more of the oil was given, which produced only a mild laxative effect, and this not until three hours after it was taken, bringing away only about thirty segments of the worm. This was on Sunday, and not wishing to keep the boy from his school, I postponed further treatment until the following Saturday, when I ordered the same amount of emulsion as before, directing that one-half of it be given in the morning, fasting, the remainder in half an hour; and one hour thereafter, ℥ xij of the liquor magnesia citratis. In a little more than two hours this occasioned active catharses; the first three dejections containing only disjointed segments of the worm, but the next brought the entire parasite, a tænia solium, twenty-two feet in length, but not dead.

I have reported this case, not as anything new, but hoping it may have some influence to induce others to test the virtues of this article. To obtain a medicine that shall be efficient for the cure of disease, at the same time that it is absolutely innocuous to the patient, is a desideratum greatly to be desired, but not always found. Of this remedy it is known that its use is neither unpleasant nor injurious, and it remains only to demonstrate its efficacy, and it is not unlikely that experience may prove it to be the best remedy for tapeworm, all things considered, that we possess.

The following are some of the advantages that the pepo may perhaps justly claim over other tænifuge remedies in common use, as the male fern, the bark of the pomegranate root, kousso, oil of turpentine, etc., 1st. To the patient it is entirely harmless and is not unpleasant to take. 2d. In this country it may always be easily obtained, and of a quality known to be good. 3d. To us it is indigenous, and other things being equal, such remedies should be preferred before those which are imported, especially in times like the present, when the whole nation should husband every resource, however trifling, for martial purposes.

Some recommend that the remedy be given in large quantities, and during a number of days, and without the adjuvant of a cathartic, for the emulsion itself when thus administered acts as a laxative; but we think an active cathartic should never be omitted, for its operation is capable of expelling a worm enfeebled by the effects of the medicine, but not to a degree to insure its death if left in the alimentary canal. It may be proper to say, that among the people who have experience with domestic remedies, there is an opinion somewhat prevalent, that the pumpkin seed is an efficient remedy for the destruction of the ascarides lumbricoides, but I am not aware that this has been confirmed by the observation of the profession.

